# Enhanced Clathrin-Dependent Endocytosis in the Absence of Calnexin

**DOI:** 10.1371/journal.pone.0021678

**Published:** 2011-07-01

**Authors:** Hao-Dong Li, Wen-Xin Liu, Marek Michalak

**Affiliations:** Department of Biochemistry, University of Alberta, Edmonton, Alberta, Canada; Ecole Polytechnique Federale de Lausanne, Switzerland

## Abstract

**Background:**

Calnexin, together with calreticulin, constitute the calnexin/calreticulin cycle. Calnexin is a type I endoplasmic reticulum integral membrane protein and molecular chaperone responsible for the folding and quality control of newly-synthesized (glyco)proteins. The endoplasmic reticulum luminal domain of calnexin is responsible for lectin-like activity and interaction with nascent polypeptide chains. The role of the C-terminal, cytoplasmic portion of calnexin is not clear.

**Methodology/Principal Findings:**

Using yeast two hybrid screen and immunoprecipitation techniques, we showed that the Src homology 3-domain growth factor receptor-bound 2-like (Endophilin) interacting protein 1 (SGIP1), a neuronal specific regulator of endocytosis, forms complexes with the C-terminal cytoplasmic domain of calnexin. The calnexin cytoplasmic C-tail interacts with SGIP1 C-terminal domains containing the adaptor complexes medium subunit (Adap-Comp-Sub) region. Calnexin-deficient cells have enhanced clathrin-dependent endocytosis in neuronal cells and mouse neuronal system. This is reversed by expression of full length calnexin or calnexin C-tail.

**Conclusions/Significance:**

We show that the effects of SGIP1 and calnexin C-tail on clathrin-dependent endocytosis are due to modulation of the internalization of the receptor-ligand complexes. Enhanced clathrin-dependent endocytosis in the absence of calnexin may contribute to the neurological phenotype of calnexin-deficient mice.

## Introduction

Calnexin is a type I endoplasmic reticulum (ER) integral membrane protein with a single transmembrane helix followed by a cytoplasmic negatively charged C-terminal tail [1], [2]. The ER luminal domain of calnexin is responsible for lectin-like activity and interaction with nascent polypeptide chains [3], [4]. Calnexin, together with calreticulin, constitute the calnexin/calreticulin cycle responsible for the folding and quality control of newly-synthesized (glyco)proteins [5], [6]. Calnexin has also been implicated in influencing Ca^2+^ regulation [1], [7], [8], [9], cell-cell adhesion [10], phagocytosis [11], and cell sensitivity to apoptosis [12], [13]. X-ray crystallography studies have identified the ER luminal domain of calnexin as forming a globular β-sandwich containing a glucose binding site with a proline-rich portion of the protein forming an extended arm structure [14], a docking site for oxidoreductase ERp57 [15], [16], [17], [18]. The C-terminal portion of calnexin extends 86 amino acid residues but its significance to calnexin function is not clear. This C-terminal domain of calnexin undergoes protein kinase-dependent phosphorylation [19], [20] and this modification may play a role in the chaperon function of the protein [20].

Endocytosis is a process by which cells internalize materials into the cell by engulfing them with plasma membrane. One sub-type of endocytosis, clathrin-dependent endocytosis, is responsible for the internalization of specific molecules into the cell including: pathogens, nutrients, antigens, growth factors and receptors [21], [22]. During clathrin-dependent endocytosis, molecules are taken into the cell by specific receptor-ligand interactions. Integral membrane proteins bind to cytosolic adaptors (AP-2), which form a link to the cytoplasmic clathrin lattice. Accessory proteins, such as Eps15 and AP180, have the ability to affect the process of clathrin mediated endocytosis by facilitating the assembly of clathrin-coated pits [23], [24], [25]. Although single-cell organisms use endocytosis as the means to obtain nourishment, higher organisms have adapted endocytosis for specialized functions. These functions include modulating interactions between signaling molecules and their receptors and providing a localized environment where signaling takes place [26], [27]. Endocytosis is especially vital for the neuronal system [28]. These processes allows retrieved fused vesicles to refill the vesicle pool allowing sustained synaptic transmission and maintenance of terminal nerve size [29]; specific substances to cross the blood-brain barrier and enter the central nervous system [30]; and internalizes receptor-ligand complexes to nerve terminals for their intracellular signaling [31]. Recent studies indicate that Src homology 3-domain growth factor receptor-bound 2-like (Endophilin) interacting protein 1 (SGIP1) may play a role in modulation of endocytotic activity in neuronal cells [32], [35].

## Results and Discussion

### The carboxyl-terminal cytoplasmic tail of calnexin interacts with SGIP1

**Figure 1:**
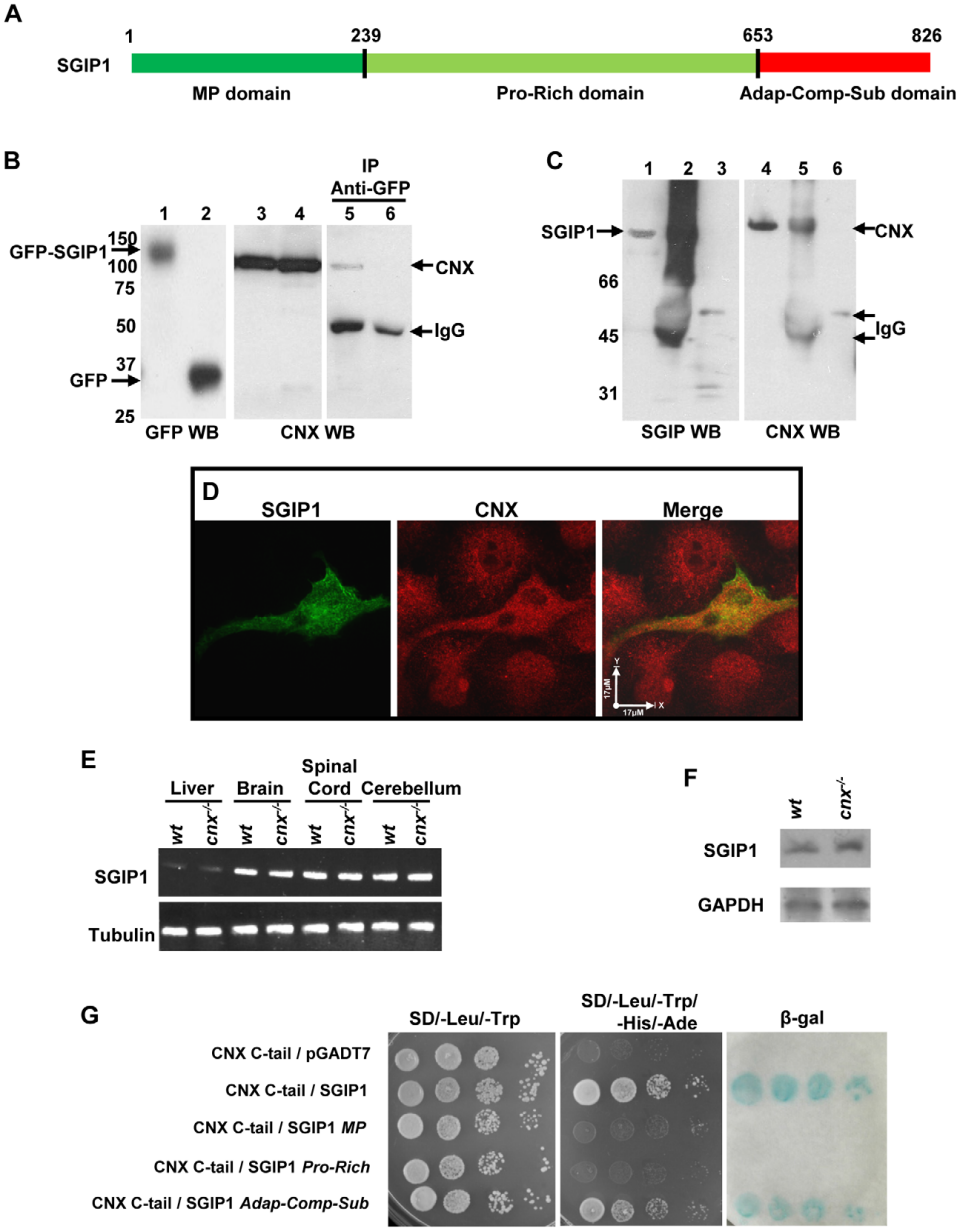
Calnexin interacts with SGIP1. (**A**) A schematic representation of full length SGIP1. The N-terminal domain of SGIP1 (amino acid residues 1–239) represents a membrane phospholipid-binding domain (MP, dark green); middle domain (amino acid residues 239–653) contains a proline-rich region (Pro-Rich, light green); the C-terminal region contains an adaptor-complex-subunit (Adap-Comp-Sub, red). (**B**) N1E-115 cells were transfected with GFP-SGIP1 or GFP expression vectors and lysed cells were subjected to immunoprecipitation with anti-GFP. Calnexin was identified by Western blot (WB) analysis of imunoprecipitates probed with anti-calnexin antibodies (*lanes 3*–*6*). *Lane 1*, Western blot analysis of N1E-115 cells expressing GFP-SGIP1; *lane 2*, Western blot analysis of N1E-115 cells expressing GFP; *lane 3*, identification of calnexin in N1E-115 cells expressing GFP-SGIP1; *lane 4*, identification of calnexin in N1E-115 cells expressing GFP; *lane 5*, lysate from N1E-115 cells expressing GFP-SGIP1 were immunoprecipitated with anti-GFP antibodies followed by Western blot analysis with anti-calnexin antibodies; *lane 6*, lysate from N1E-115 cells expressing GFP were immunoprecipitated with anti-GFP antibodies followed by Western blot analysis with anti-calnexin antibodies. (**C**) Immunoprecipitation of SGIP1-calnexin complexes from mouse cerebellum. Homogenate from wild-type mouse cerebellum was incubated with anti-SGIP1 antibodies or anti-His-tag antibodies followed by Western blot analysis with either anti-SGIP1 antibodies (*lanes 1*–*3*) or anti-calnexin antibodies (*lanes 4*–*6*). *Lanes 1* and *4*, cerebellum homogenate; *lanes 2* and *5*, immunoprecipitated with anti-SGIP1 antibodies; *lanes 3* and *6*, immunoprecipitation with anti-His-tag antibodies. CNX, calnexin. (**D**) Immunolocalization of calnexin and GFP-SGIP1 in N1E-115 cells. N1E-115 cells were transfected with pEGFP-SGIP1 expression vector followed by confocal analysis of intracellular localization of GFP-SGIP1 and calnexin as described under “Experimental Procedures”. Scale bar = 17 µm. (**E**) mRNA were isolated from liver, brain, spinal cord and cerebellum of wild-type (*wt*) and calnexin-deficient (*cnx* ^−*/*−^) mice followed by RT-PCR with DNA primers specific for SGIP1 (*SGIP1*) or tubulin (*tubulin*) as described under “Experimental Procedures”. (**F**) Western blot analysis of protein extracts isolated from wild-type (*wt*) and calnexin-deficient (*cnx* ^−*/*−^) granule cells was carried out with anti-SGIP1-antibodies as described under “Experimental Procedures”. (**G**) Yeast cells were transformed with pGBKT7 vector containing cDNA encoding calnexin C-tail (*CNX C-tail*) and pGADT7 vector or pGADT7 vector containing cDNA encoding SGIP1 or SGIP1 domains as depicted in the Figure. Yeast serial dilution-culture (10^−1^∼10^−4^) on SD/-Leu/-Trp plate shows both pGBKT7 and pGADT7 vectors were transformed into the AH109 strain. Yeast culture on SD/-Leu/-Trp/-His/-Ade shows yeast-two-hybrid interaction and activation of the reporter gene. Filter lift assays indicates interactions between the calnexin C-tail and SGIP1 and specifically the SGIP1 C-terminal Adap-Comp-Sub domain.

Calnexin is composed of structural and functional domains [33]. While the ER luminal region is responsible for the chaperone function of the protein, the transmembrane domain anchors the protein to the membrane and the C-terminal tail extends to the cytoplasmic compartment. To examine the functional importance of cytoplasmic tail of calnexin we carried out the yeast two hybrid screening method to identify proteins interacting with the C-terminal cytoplasmic domain of calnexin (designated C-tail) encompassing the last 88 amino acids (C^504^-E^591^) of the protein. Because calnexin-deficient mice have neurological disorders [34], [44], we used a mouse brain Matchmaker cDNA library (638841, Clontech, USA) to identify calnexin interacting proteins in a neuronal system. Screening of the library resulted in the identification of 9 positive clones. Three of these clones encoded the C-terminal region of SGIP1 (Src homology 3-domain growth factor receptor-bound 2-like (Endophilin) interacting protein 1) [32], [35]. The longest cDNA fragment of SGIP1 identified in the screen was 537 nucleotides and encoded the last 179 amino acids of the protein. This C-terminal region of SGIP1 contains the domain referred to as the adaptor complexes medium subunit (Adap-Comp-Sub) (Fig. 1A). This adaptor complex may couple clathrin lattices with membrane proteins and play a role in clathrin-dependent endocytosis (http://pfam.sanger.ac.uk/family?id=Adap_comp_sub).

To test for direct interactions between the calnexin C-tail domain and SGIP1, we carried out immunoprecipitation analysis. N1E-115 neuroblastoma cells were transfected with expression vectors encoding GFP-SGIP1 or GFP control followed by cell lyses, immunoprecipitation with anti-GFP antibodies and Western blot analysis with anti-calnexin antibodies. Figure 1B (*lane 5*) shows that calnexin was present in GFP-SGIP1 immunoprecipitate indicating that these two proteins interacted. As expected, there was no immunoreactive calnexin in GFP expressing cells (Fig. 1B, *line 6*). Since SGIP1 is predominantly expressed in the brain, especially in cerebellum ([35], http://www.ncbi.nlm.nih.gov/UniGene/ESTProfileViewer.cgi?uglist=Mm.238094), we used cerebellum extracts to test for endogenous SGIP1-calnexin complexes. Anti-SGIP1 antibodies immunoprecipitated SGIP1 from cerebellum homogenate (Fig. 1C, *lane 2*), and the complexes contained immunoreactive calnexin (Fig. 1C, *lane 5*). Since SGIP1 antibodies were generated against His-tagged recombinant SGIP1 we also employed anti-His-tag antibodies as a negative control (Fig. 1C, *lanes 3 and 6*). We concluded that calnexin interacted with SGIP1 *in vivo* in N1E-115 cells and in cerebellum. Next we carried out immunolocalization analysis in N1E-115 cells expressing recombinant GFP-SGIP1. Figure 1D shows that GFP-SGIP1 and calnexin co-localized to the ER-like network. Quantitative analysis for SGIP1-calnexin colocalization showed that 66.78% (Std. Error = 4.22%, n = 20) of GFP-colocalized with calnexin, supporting the biochemical and immunological results that the two proteins form complexes. The RT-PCR analysis showed that SGIP1 is predominantly expressed in brain, spinal cord and cerebellum (Fig. 1E). This is in agreement with earlier studies [32], [35] indicating that there is a high expression of the protein in neuronal tissues. Since calnexin-deficient mice develop neuropathies, we examined whether calnexin affects expression of SGIP1. Figures 1E and F show that expression of SGIP1 mRNA and protein was not affected in the absence of calnexin.

To carry out molecular analysis of the interaction between calnexin and SGIP1, we isolated cDNA encoding mouse SGPI1 from the mouse brain Matchmaker cDNA library followed by nucleotide sequence analysis of the clone. Nucleotide and amino acid sequences of this mouse variant of SGIP1 (2481 bp, encoding a protein of 826 amino acids) differed from the rat SGIP1α [35] and the mouse SGIP1 released from NCBI (Fig. S1). Important difference between the clones was that the region encompassing amino acid residues G35-Q62 in rat SGIP1α was not present in mouse SGIP1 (Fig. S1). Comparison of the protein sequence of mouse SGIP1 isolated in our laboratory with that available at the NCBI revealed two additional differences. The mouse clone isolated in this study had an L in position 295 versus an F in the NCBI sequence and a P in position 355 versus a T in the NCBI sequence (Fig. S1). We concluded that the calnexin C-terminal tail interacts with SGIP1, a newly identified neuronal version.

MP domain of SGIP1 binds phospholipids and it may be involved in interactions with Esp15 [32], an adaptor protein of clathrin-mediate endocytosis [22]. The central, proline-rich domain of SGIP1 may form complexes with endophilin-3 [35], an important regulator of clathrin-mediated endocytosis and synaptic vesicles recycling [21], [22]. Next we mapped the SGIP1 domains interacting with the calnexin C-tail. To do this we generated cDNA encoding three different regions of SGIP1 (Fig. 1G): the N-terminal domain membrane phospholipid-binding domain (MP, amino acid residues 1–239) and a proline rich region (Pro-Rich, amino acid residues 239–653), and a domain containing the adaptor complexes medium subunit (Adap-Comp-Sub, amino acid residues 647–826), and cloned into the pGADT7 vector to test their interaction with the calnexin C-tail in the yeast-two-hybrid system. Yeast-two-hybrid analysis revealed that calnexin-C-tail interacted with the C-terminal region of SGIP1 (Adap-Comp-Sub domain, amino acid residues 647–826, Fig. 1G). We concluded that interaction between the calnexin C-domain and SGIP1 maps to the Adap-Comp-Sub domain of SGIP1.

### Increased clathrin-dependent endocytosis in the absence of calnexin

**Figure 2:**
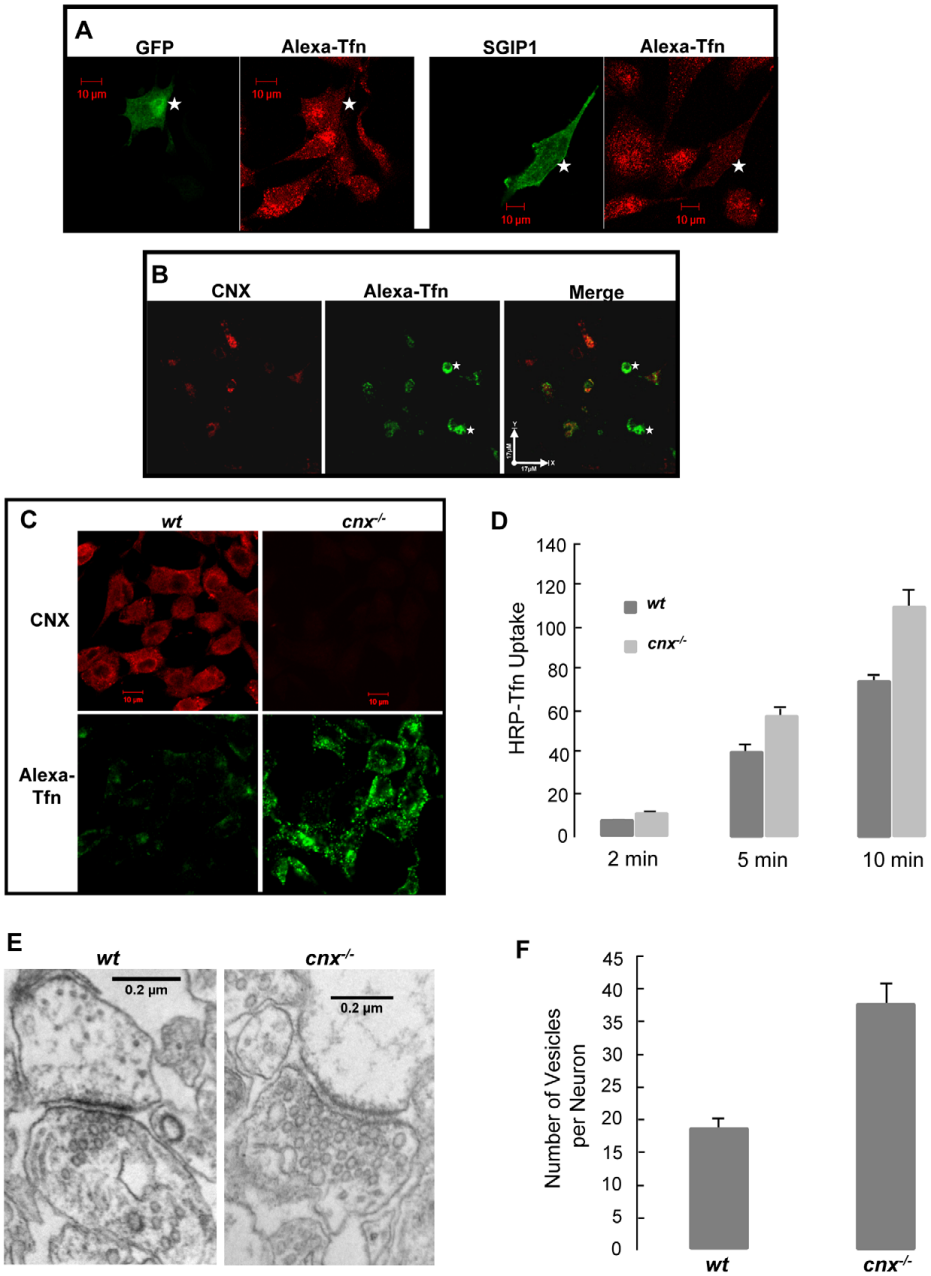
Increased endocytosis in the absence of calnexin. (**A**) Alexa647-transferrin (Alexa-Tfn) uptake in N1E-115 cells expressing GFP (*left panels*) or GFP-SGIP1 (*right panels*). Non-transfected N1E-115 cells or N1E-115 cells expressing GFP (*left panels*) show significant uptake of Alexa-Tfn. In contrast cells expressing GFP-SGIP1 (*right panels*) have significantly inhibited Alexa-Tfn uptake. The asterisks indicate the location of cells expressing GFP or GFP-SGIP1. Scale bar = 10 µm. (**B**) Alexa-Tfn endocytosis in a mixed culture of granule cells isolated from wild-type and *cnx* ^−*/*−^ mice. *Left panel*, immunostaining with anti-calnexin antibodies. *Middle panel*, granule cells were incubated with Alexa-Tfn at 37°C for 10 min followed by confocal microscopy. *Right panel*, a merge of both images. The asterisks indicate the location of calnexin-deficient cells. Scale bar = 17 µM. (**C**) Confocal images of wild-type (*wt*) and calnexin-deficient (*cnx* ^−*/*−^) fibroblasts incubated with Alexa-Tfn at 37°C for 10 min. Calnexin was identified with anti-calnexin antibodies. Scale bar = 10 µm. (**D**) Quantitative analysis of HRP-Tfn uptake of wild-type *(wt*) and calnexin-deficient (*cnx* ^−*/*−^) fibroblasts. HRP-Tfn was incubated with fibroblasts at 37°C for the time indicated in the Figure. (**E**) Electron microscope analysis of clathrin-coated synaptic vesicles in neurons of wild-type (*wt*) and calnexin-deficient (*cnx* ^−*/*−^) cerebellum. Scale bar = 0.2 µm. (**F**) Quantitative analysis of synaptic vesicles in neurons of wild-type (*wt*) and calnexin-deficient (*cnx* ^−*/*−^) mice. Vesicles were counted in sixteen neurons for the wild-type, and ten neurons for the calnexin-deficient (*cnx* ^−*/*−^) mice.

Considering that SGIP1 is a neuronal endocytotic protein interacting with adaptor proteins involved in clathrin-dependent endocytosis [32], [35] and that the SGIP1α has been implicated to play a role in endocytosis in neuronal cells [32], we tested whether calnexin may play any role in neuronal endocytosis. First, we examined whether GFP-SGIP1 affects uptake of transferrin in N1E-115 cells. Transferrin has been commonly used as an indicator of clathrin-dependent endocytosis [36]. N1E-115 cells were transfected with either GFP-SGIP1 or GFP expression vectors, incubated with Alexa-transferrin, fixed and examined by confocal microscopy. As expected, expression of GFP in N1E-115 cells did not have any effect on transferrin uptake (Fig. 2A, *left panel*). In contrast, GFP-SGIP1 expressing cells had significantly inhibited transferrin uptake (Fig. 2A, *right panel*).

Second, we asked whether clathrin-dependent endocytosis might also be affected in the absence of calnexin. Cerebellar granule cells were isolated from of 7 day old wild-type and calnexin-deficient mice. A mixed granule cell culture was established containing wild-type and calnexin-deficient cells. Cells were cultured for 4 days in neurobasal plus medium followed by the transferrin uptake assay. Wild-type cells were identified by a positive staining with anti-calnexin antibodies (Fig. 2B, *left panel*). Analysis of transferrin uptake of mixed cultures showed significantly increased transferrin uptake in all calnexin-deficient cells examined (n = 80) (Fig. 2B, compare *middle* and *left panels*) indicating that there was increased clathrin-dependent endocytosis in granule cells isolated from calnexin-deficient mice. In addition to granule cells, increased transferrin uptake was also observed in calnexin-deficient fibroblasts (Fig. 2C and 2D) indicating that there was significant increase in endocytic activities in cells lacking calnexin.

Next we carried out electron microscopy analysis of cerebella from 7 day old wild type and calnexin-deficient mice to examine whether there was increased clathrin-dependent endocytosis in mice in the absence of calnexin mice. Figure 2E shows that calnexin-deficient mice had significantly increased number of synaptic vesicles when compared to wild-type cerebellum. The numbers of synaptic vesicle were quantitated using 16 wild-type neurons and 10 calnexin-deficient neurons. The mean number of clathirn coated vesicles for wild-type and calnexin-deficient neurons was 18.6±1.5 and 37.3±3.6, respectively (Fig. 2F). We concluded that calnexin deficiency leads to increased endocytotic activity in cultured cells and mouse neuronal system.

**Figure 3:**
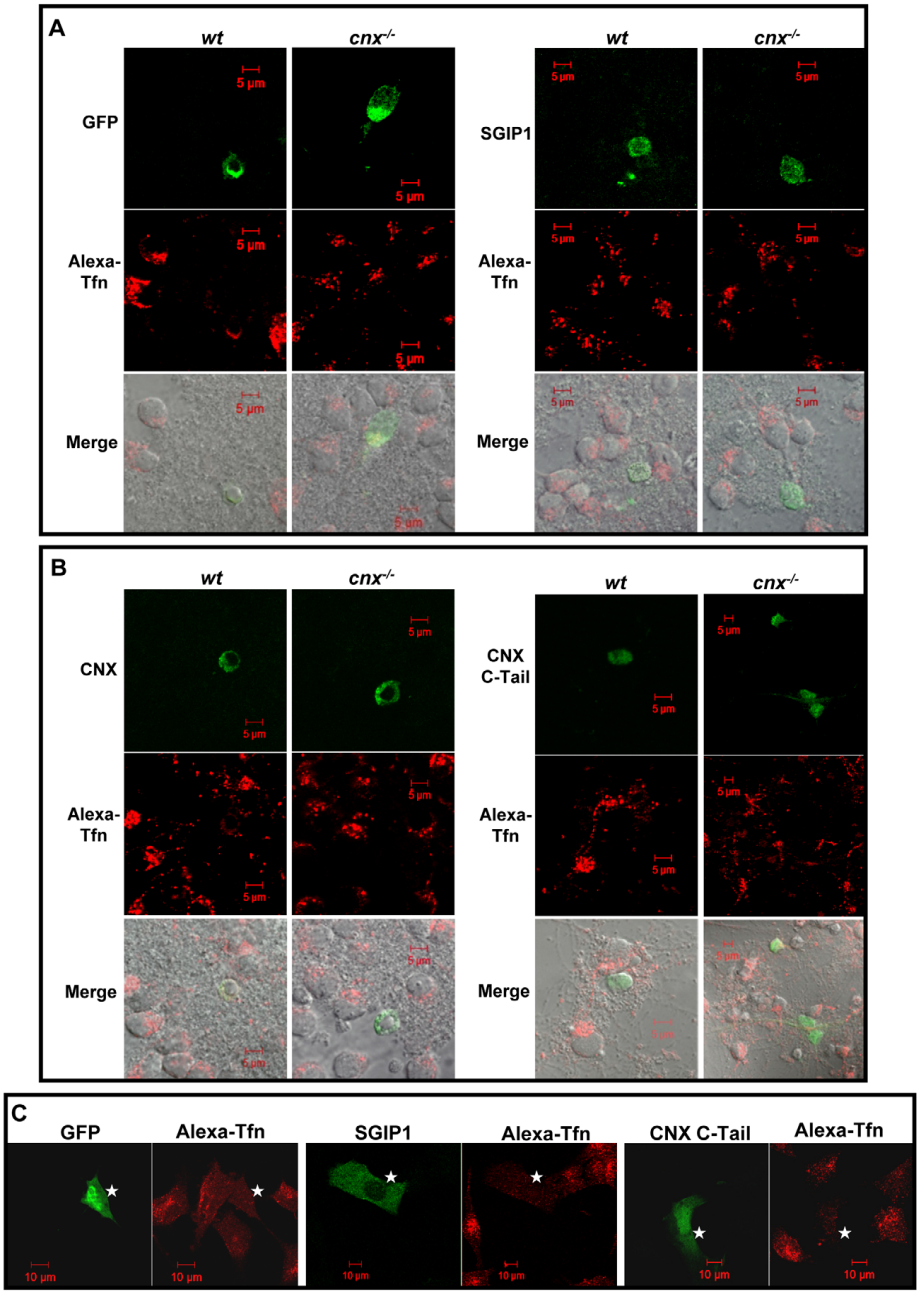
Calnexin C-tail inhibits transferrin uptake. (**A**) Alexa647-transferrin (Alex-Tfn) uptake in wild-type (*wt*) and calnexin-deficient (*cnx* ^−*/*−^) granule cells expressing GFP (*left panel*) or GFP-SGIP1 (*right panel*). Scale bar = 5 µm. (**B**) Alexa-Tfn endocytosis in wild-type (*wt*) and calnexin-deficient (*cnx* ^−*/*−^) granule cells expressing GFP tagged full-length calnexin (*left panel*) or GFP tagged C-tail of calnexin (CNX C-Tail) (*right panel*). Granule cells were incubated with Alexa-Tfn at 37°C for 10 min as described under “Experimental Procedures”. Scale bar = 5 µm. (**C**) N1E-115 neuronal cells were transfected with expression vectors encoding GFP, GFP tagged SGIP1 (SGIP1) or calnexin C-tail (CNX C-Tail) followed by Alexa-Tfn uptake analysis. N1E-115 cells expressing GFP were used as control. The asterisks indicate cells expressing recombinant GFP-SGIP1 or recombinant calnexin C-tail (CNX C-Tail). Scale bar = 10 µm.

Next, we examined whether expression of full length calnexin in *cnx* ^−*/*−^ cells will reduce the endocytotic activities seen in calnexin-deficient cells. We isolated granule cells from wild-type and calnexin-deficient cerebellum, followed by expression of recombinant GFP, GFP-SGIP1, full length calnexin or calnexin C-tail. Expression of GFP alone in wild-type and calnexin-deficient cells had no effects on transferrin uptake which remained high as observed for calnexin-deficient cells (Fig. 3A, *left panel*). As expected, expression of SGIP1 had an inhibitory effect on endocytosis in granule cells independent of the presence (*wild-type*) or absence (*cnx* ^−*/*−^) of calnexin (Fig. 3A, *right panel*). Figure 3B (*left panel*) shows that both wild-type and calnexin-deficient cells transfected with calnexin expression vector and expressing full-length calnexin had reduced transferrin uptake compared to control, non-transfected cells. Transferrin uptake in these cells was similar to that observed for wild-type cells. Combined results presented in Figures 2 and 3 indicate that that calnexin and SGIP1 affected endocytotic activities of cerebella granule cells. Since we observed that the C-terminal domain of calnexin (C-tail) interacted with SGIP1, we asked whether the C-tail of calnexin alone would have any effect on transferrin uptake in granule cells. Figure 3B (*right panel*) shows that expression of recombinant, GFP tagged C-tail of calnexin resulted in significantly reduced endocytosis in calnexin-deficient and wild-type granule cells. We also tested for the role of SGIP1 and calnexin C-tail in endocytosis in the N1E-115 neuronal cell line. Figure 3C shows, that similar to granule cells, expression of SGIP1 and calnexin C-tail in N1E-115 cells resulted in reduced transferrin uptake indicative of reduced endocytosis.

**Figure 4:**
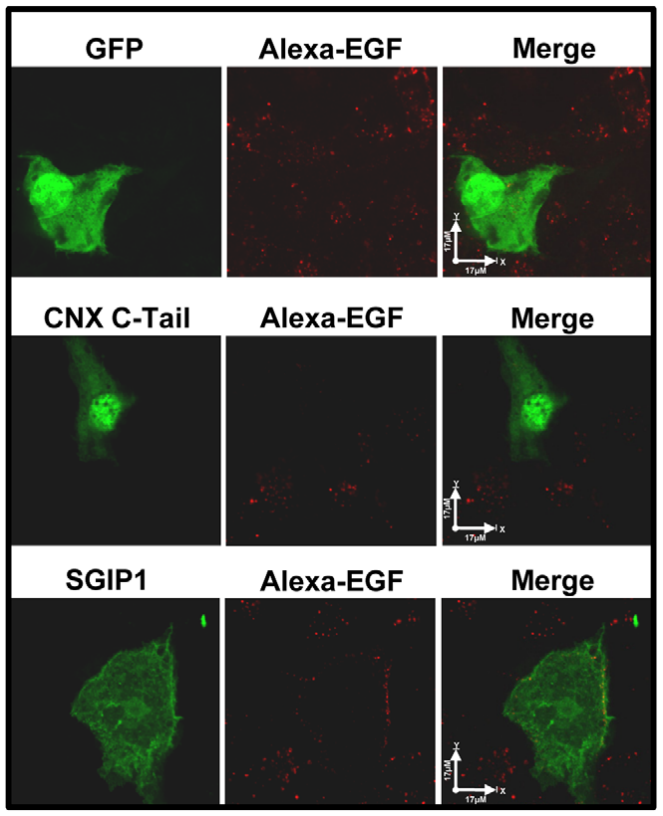
Calnexin affects clathrin-dependent endocytosis. N1E-115 cells were transfected with expression vectors encoding GFP, GFP tagged SGIP1 (SGIP1) or calnexin C-tail (CNX C-Tail) and incubated with Alexa647-Epidermal Growth Factor (Alexa-EGF), a ligand-induced clathrin-dependent endocytosis marker at 37°C for 10 min. Scale bar = 17 µm.

Next, we tested whether epidermal growth factor (EGF) as another marker for clathrin-dependent endocytosis was affected by calnexin in neuronal cells. N1E-115 neuronal cells were transfected with expression vectors encoding GFP-calnexin C-tail, or GFP-SGIP1 followed by analysis of EGF induced endocytosis. Figure 4 shows that expression of calnexin C-tail or SGIP1 in N1E-115 cells significantly decreased Alexa-EGF uptake in all examined cells that expressed GFP fusion proteins (n = 90) indicating that calnexin C-tail and SGIP1 inhibited clathrin-dependent endocytosis. These findings indicate that calnexin C-tail is an important factor involved in clathrin-dependent endocytosis. It is likely that the calnexin C-tail exerts its effects on endocytosis by interacting with SGIP1, a protein that forms complexes with endophilin-3 [35], AP-2 complex, Eps15 and phospholipids [32] to affect clathrin-dependent endocytosis.

### Calnexin C-tail affects endocytosis at ligand internalization

**Figure 5:**
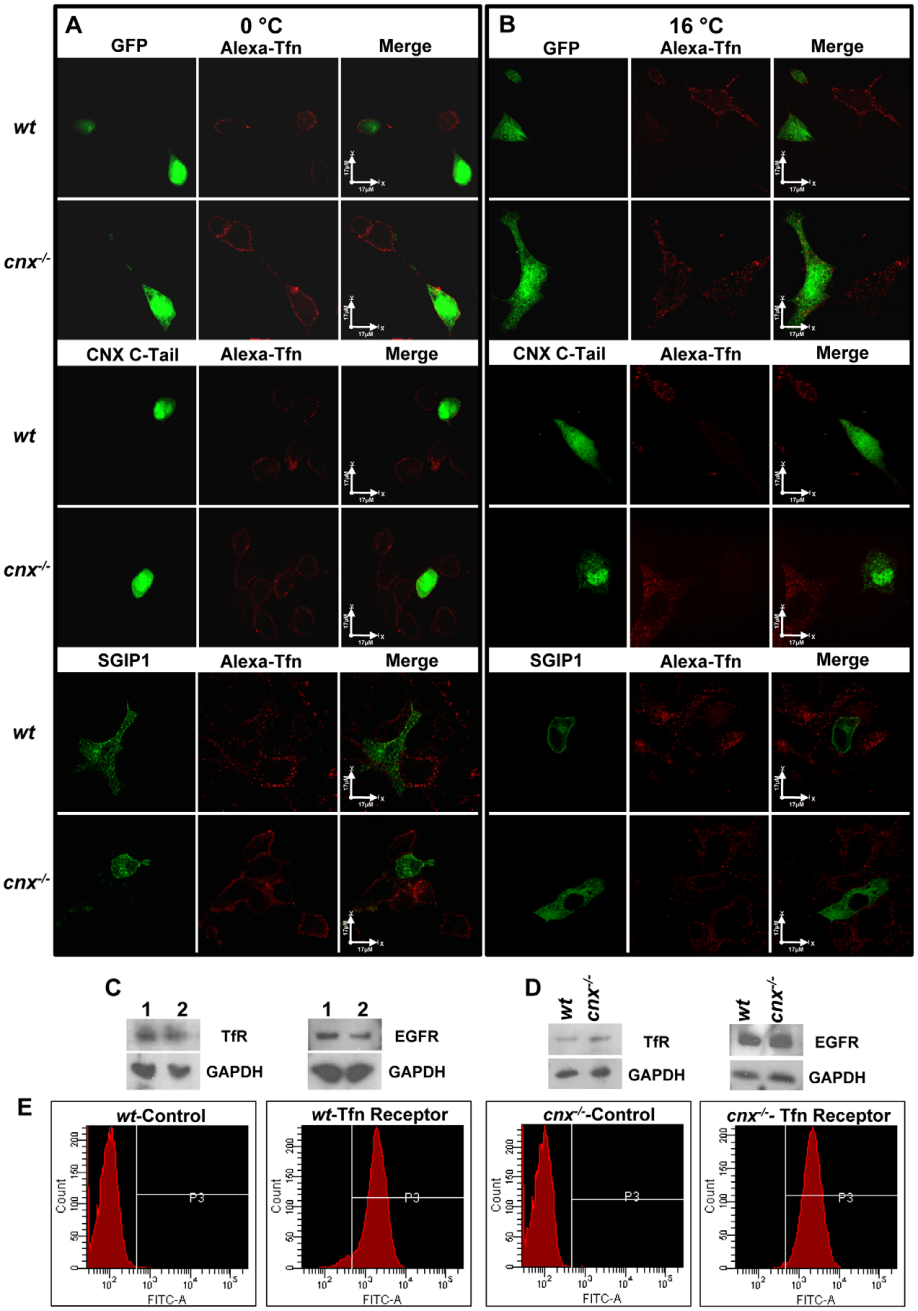
Calnexin affects ligand-internalization step in clathrin-dependent endocytosis. Wild-type (*wt*) and calnexin-deficient (*cnx* ^−*/*−^) fibroblasts were transfected with expression vectors encoding GFP, GFP tagged calnexin C-tail (CNX C-Tail), or GFP tagged SGIP1 (SGIP1). Cells were incubated with Alexa647-transferrin (Alexa-Tfn) on ice for 60 min at 0°C (**A**) for 30 min at 16°C (**B**). Scale bar = 17 µm. (**C**) Western blots analysis of N1E-115 cells expressing GFP-calnexin C-tail (*lane 1*) or GFP *(lane 2*). Blots were probed with anti-transferrin receptor (TfR) antibodies, anti-epidermal growth factor receptor (EGFR) antibodies and anti-glyceraldehyde-3-phosphate dehydrogenase (GADPH) antibodies. (**D**) Western blots analysis of cerebella from wild type mouse (*wt*) and calnexin-deficient mouse (*cnx* ^−*/*−^). Blots were probed with anti-transferrin receptor (TfR) antibodies, anti-epidermal growth factor receptor (EGFR) antibodies and anti-glyceraldehyde-3-phosphate dehydrogenase (GADPH) antibodies. (**E**) Wild-type (*wt*) and calnexin-deficient (*cnx* ^−*/*−^) cells were analyzed flow cytometry with anti-Transferrin receptor antibodies. Results are presented as the relative mean florescence intensity after subtracting unspecific staining. P3 represents the gate set on cells stained with antibody.

Clathrin-dependent endocytosis is a temperature sensitive process. At physiological temperature (37°C) there is rapid clathrin-dependent endocytosis, whereas at 16°C, endocytosis is slow and permissive for receptor-ligand internalization and does not support recycling or degradation [37]. In contrast, at 0–4°C, internalization of the ligand is inhibited without any effect on ligand binding to the receptor [37]. We took advantage of these temperature-dependent effects on endocytosis to examine which phase of clathrin-dependent endocytosis was affected by SGIP1 and the calnexin C-tail. To do this, N1E-115 cells expressing recombinant GFP-SGIP1 or GFP calnexin C-tail were incubated at different temperatures (4°C or 16°C) followed by analysis of Alexa-transferrin uptake. Transferrin ligand binding to cells expressing either GFP-SGIP1 or GFP-calnexin C-tail was not affected at 0°C (Fig. 5A). However, there was no internalization of the ligand under these conditions in all cells that expressed GFP fusion proteins (n = 90) (Fig. 5A). In contrast, at 16°C, all cells expressing recombinant GFP-SGIP1 or GFP-calnexin C-tail (n = 90) had reduced Alexa-transferrin uptake compared to GFP expressing cells or non-transfected control cells (Fig. 5B). Since receptor recycling and degradation are inhibited at 16°C [37], we concluded that SGIP1 and the calnexin C-tail affected the ligand-internalization step during clathrin-dependent endocytosis. Western blot analysis revealed that expression of transferrin and EGF receptors was not affected in cells expressing calnexin C-tail (Fig. 5C). And there's no differences between wild type (*wt*) and calnexin-deficient (*cnx* ^−*/*−^) cerebella on Transferrin or EGF receptors expression level either (Fig. 5D). Next, we carried out FACS analysis to quantify the transferrin receptor levels in wild-type and *cnx* ^−*/*−^ cell lines. Figure 5E shows that the level of cell surface expression of transferrin receptor was the same in wild-type and and *cnx* ^−*/*−^ cell lines. These results supported a notion that the effects of SGIP1 and calnexin C-tail on clathrin-dependent endocytosis were not due to a reduced level of transferrin or EGF receptors, but were due to modulation of the internalization of the receptor-ligand complexes.

Although many fundamental eukaryotic trafficking mechanisms are conserved in neurons, they have evolved distinct modes of trafficking to accommodate their unique morphology and function [37]. Furthermore, different endocytotic mechanisms specific for different surface molecules and cellular domains exist in neurons [28]. Specificity of calnexin to affect clathrin-dependent endocytosis in the nervous system is remarkable and likely dictated by calnexin interaction with SGIP1. The N1E-115 neuronal cell line has very low SGIP1 expression and does not express Eps15 or AP180, and full length calnexin does not affect endocytosis in these cells (Fig. S2). In sharp contrast, cultured granule cells expressing large quantities of SGIP1, are highly sensitive to calnexin-dependent effect on endocytosis (Fig. 2B and Fig. 3B). Neurons are specialized types of cells, and the exocytosis-endocytosis balance is vital for neurons to play its normal functions and to survive. In addition, there are specific protein components required for clathrin-dependent endocytosis in neurons, for example, neuron-specific variants of α- and β-adaptin chains have been identified [38]. We found that SGIP1 is primarily expressed in the neuronal system, and other essential neuronal endocytic factors, AP180 and Eps15, were also more abundant in the cerebellum than in neuronal cell lines (Fig. S2). Association of specific endocytosis related factors with nerve terminal-derived vesicles suggests a functional specialization of nerve terminal to adapt to the specific requirements of synaptic vesicle recycling [39]. The specific intra- and intercellular environment of the neuron may provide factors that cooperate with full length calnexin to decrease clathrin-dependent endocytosis.

One of the best described functions of calnexin is its role as a molecular chaperone [40]. Little information is available about the structure and function of the cytoplasmic calnexin C-tail as the majority of studies on calnexin has focused on its ER luminal lectin-like chaperon domain [41]. Here we showed that the calnexin C-tail is a potent inhibitor of endocytosis in neuronal cells indicating a functional specialization of calnexin domains. The C-terminal region of calnexin binds Ca^2+^ and can be phosphorylated by specific protein kinases and this enhances calnexin's interactions with ribosomes [19], [20]. Ca^2+^ may affect endocytosis by the neuronal system by arresting synaptic vesicle movement or increasing synaptic vesicle size [42], [43]. Absence of calnexin results in a severe neurological phenotype in mice manifested by impaired gait, reduced lower limb function [34], [44] and inhibited nerve conductive velocity due to impaired myelination of the peripheral and central nervous system [34]. Increased endocytosis in the nervous system in the absence of calnexin as described in this study may also, at least in part, contribute to the phenotype of calnexin-deficient mice. For example, endocytosis and postendocytic sorting of neurotransmitter receptors have emerged as critical mechanisms responsible for various forms of synaptic plasticity [28]. Calnexin may affect these critical pathways and impact synaptic plasticity and consequently the function of the nervous system. This, at least in part, might be mediated by C-terminal cytoplasmic domain of calnexin.

## Materials and Methods

### Plasmids, yeast two-hybrid assays and RT-PCR

The mouse brain Matchmaker cDNA library in pACT2 (Clontech, 638841) was transformed into yeast strain AH109. The library was screened for interacting proteins with the C-terminal cytoplasmic region of calnexin (C-tail) corresponding to the last 87 amino acids (C504-E591). cDNA encoding the C-tail of calnexin was obtained by PCR amplification of a mouse calnexin template cDNA using the following primers: the 5′ forward primer 5′-GGA ATT CCA TAT GTG TTC TGG AAA GAA AC-3′ and the 3′ reverse primer 5′-AAG GTT CTG CAG TCA CTC TCT TCG TGG CT-3′. The PCR product was cloned in frame with the GAL4 DNA-binding domain at *Nde*I and *Pst*I restriction sites to generate the clone pGBKT7-CNX-C. Screening of the mouse library was carried out as recommended by the manufacturer (Clontech). Plasmid DNA from positive clones was isolated and used as template for amplification of the insert in the library vector by the AD (activating domain) sequencing primer and T7 Y2H sequencing primer.

For synthesis of cDNA encoding SGIP1, RNA was isolated from liver, brain, spinal cord and cerebellum using TRIzol (Invitrogen). For PCR-driven synthesis of cDNA encoding SGIP1 the following primers were used: forward primer 5′-CGC GGA TCC GAA TGA TGG AAG GAC TGA AA-3′ and reverse primer 5′-CCG CTC GAG TTA GTT ATC TGC CAA GTA CT-3′. For RT-PCR the following primers were used: for SGIP1 forward primer 5′-AAT GTG GAC ATG CTC AA ATA-3′ and reverse 5′-TTA GTT ATC TGC CAA GTA CT-3′; for tubulin forward 5′-CCG GAC AGT GTG GCA ACC AGA TCG G-3′, and reverse 5′-TGG CCA AAA GGA CCT GAG CGA ACG G-3′.

### Cell culture, immunoprecipitation, Western blot and FACS analyses

Cerebellum was isolated from a 7-day-old mouse, cut into small pieces and digested in trypsin for 12 min at 37°C. Tissues was washed twice with PBS and spun down at 350 x*g* for 5 min to collect dissociated cells. Cells were suspended in neurobasal medium (Gibco) with 2% B-27, 80 mg/L D-glucose, 20 µM l-glutamine, 1% Penicillin/Streptomycin and 20 mM KCl, and then passed through a 40 µm filter. Granule cells (5×10^6^ freshly isolated granule cells) were combined with 2 µg plasmid and 100 µL mouse neuron nucleofector (Amaxa). Transfection of cell lines with expression vectors was carried out using the lipofectamine 2000 system (Invitrogen) according to the manufacturer's recommendation.

For immunoprecipitation, N1E-115 neuronal cells (CRL 2263 from the American Type Culture Collection) were treated for 15 min on ice with RIPA buffer containing 50 mM Tris, pH 7.4, 150 mM NaCl, 1% NP-40 (nonyl phenoxylpolyethoxylethanol), 0.25% sodium deoxycholate and 1 mM EDTA. For cerebellum immunoprecipitation, cerebellum was grinded into powder in liquid nitrogen followed by incubation with a RIPA buffer. Solubilized cells or cerebellum tissue power was spun down at 16,000 xg for 10 min at 4°C. Sample was pre-cleared by incubation with 30 µL protein and A/G-Sepharose beads for 30 min. Antibodies were added to pre-cleared sample and incubated at 4°C for 3 hrs on an orbital rocker. Thirty µL of Protein A/G bead solution was added followed by overnight incubation at 4°C. Beads were spun down at 1,000 xg for 3 min, washed 5 times with ice cold PBS and re-suspend in SDS-PAGE sample buffer. Proteins were separated by SDS-PAGE (7.5% acrylamide) followed by Western blot analysis with goat anti-GFP (1∶10,000, Abcam), rabbit anti-calreticulin (1∶1,000, Abcam), rabbit anti-calnexin (1∶1,000, Stressgen Bioreagents), rabbit anti-transferrin receptor (1∶200, Santa Cruz Biotechnology), rabbit anti-EGFR (epidermal growth factor receptor) (1∶500, Santa Cruz Biotechnology), mouse anti-AP180 (1∶200, Sigma) and mouse anti-Eps15 (1∶200, BD Biosciences), rabbit anti-SGIP1 (1∶200, homemade). For SGIP1 antibody generating, we cloned coding sequence of last 159 amino acids from mouse and it was clone in frame with 6×His Tag at *Xho*I and *Kpn*I restriction sites of pBAD/gIII-A vector to express SGIP1 C-terminus-His Tag peptide in *E. coli.* strain Rosetta BL21 (Novagen, Germany). SGIP1 C-terminus-His was purified by Ni^2+^-affinity chromatography. New Zealand white rabbits (2∼2.5 kg) was injected with 0.5 mg SGIP1 CT-His mixed with 0.5 mL Freund's adjuvant (Sigma, F-5506) three times before collect blood to harvest antiserum. Before injection, 2 mL bleed was taken from the rabbit for pre-bleed serum. Protein concentration was estimated using a BioRad DC Protein assay [45]. All animal experimental procedures were approved by the Animal Welfare Program at the Research Ethics Office, the Health Sciences Animal Policy and Welfare Committee (ID 069), University of Alberta and conformed to the guidelines set forth by the Canadian Council on Animal Care.

FACS analysis was carried out using BD FACScan single laser flow cytometry (BD Bioscience) equipped with a 488-nm filter. Data were collected from 10,000 cells and analyzed using CellQuest software. Cells at 80–90% confluence were harvested by scraping into PBS, re-suspended in 100 µL of 0.1% formaldehyde in PBS, and incubated for 30 min with the anti-transferrin receptor antibodies (eBioscience, 11–0711). Cells stained with FITC alone were used as negative control.

### Endocytosis analysis

Prior to endocytosis analysis cells were starved in serum-free medium for 1 hr followed by incubation with 25 µg/mL transferrin (Tfn) Alexa-Fluor 647 and 100 ng/mL EGF Alexa-Fluor 647 at 37°C for 10 min. Ligand uptake was terminated by placing cells on ice. Cells were rinsed in PBS and fixed in 3.7% paraformaldehyde. Internalized ligand was visualized using confocal microscopy. For Tfn endocytosis quantification, cells were first incubated with 25 µg/mL HRP-Tfn on ice for 60 min, and then shifted to 37°C for 2 min, 5 min or 10 min. Cells were washed twice with cold PBS/30 mM glycine at pH 2.7, twice with ice cold PBS and lysed with 1% Triton X-100 in PBS. Cells were lysed and 200 µL lysate aliquots were missed with 1 mL SIGMAFAST™ OPD (o-phenylenediamine dihydrochloride) solution (Sigma) for HRP-OPD analysis to reveal HRP-Tfn uptake. The HRP-OPD color developing reaction was stopped by addition of 1/4 volume of 3 M H2SO4 followed by OD492 measurements.

### Immunofluorescence, confocal and electron microscopy

Immunofluorescence and confocal microscopy was carried out as described previously [46]. Both rabbit anti-calnexin and goat anti-rabbit Alexa secondary antibody were diluted as 1∶200 in PBS plus 2% milk powder and 0.1% saponin. All confocal microscopy images were taken under 60× objectives.

For electron microscopy analysis, 7-day old mice were euthanized by decapitation and their cerebella were taken. Primary fixation was carried out at 4°C for 4 hrs in a freshly prepared solution of 2.5% glutaraldehyde and 2% paraformaldehyde in 100 mM cacodylate buffer pH 7.2, and then fixed in 2.5% glutaraldehyde, 100 mM sodium cacodylate pH 7.0 [47]. Samples were processed for electron microscopy and examined with a Hitachi Transmission Electron Microscope H-7000.

## Supporting Information

Figure S1 **Comparison of amino acid sequence of different SGIP1 isoforms.** Protein sequence alignment for mouse SGIP1 sequence gotten from this study (SGIP1), mouse SGIP1 sequence release from NCBI (SGIP1-NCBI; GenBank: CAM14981) and rat SGIP1 alpha sequence shown in ref. 32 (SGIP1-alpha; GenBank:AB262964).
(EPS)

Figure S2 **Expression of endocytic proteins in the absence of calnexin.** (**A**) Expression of full length calnexin did not inhibit endocytosis in N1E-115 cells. Alexa 647-transferrin (Alex-Tfn) uptake in N1E-115 cells expressing GFP (*upper panel*) or full length calnexin (CNX, *lower panel*). (**B**) Western blot analysis of extracts from N1E-115 cell line and cerebellum was carried out with anti-calnexin, anti-SGIP1, anti-Eps15 and anti-AP180 antibodies. Anti-GAPDH antibodies were used as a loading control.
(EPS)
